# Treatment of stage I seminoma: is it time to change your practice?

**DOI:** 10.1186/1756-8722-1-22

**Published:** 2008-11-07

**Authors:** Darren R Feldman, George J Bosl

**Affiliations:** 1 Assistant Attending, Genitourinary Oncology Service, Department of Medicine, Memorial Sloan-Kettering Cancer Center, USA; 2 Chair, Department of Medicine, The Patrick M. Byrne Chair in Clinical Oncology, Department of Medicine, Memorial Sloan-Kettering Cancer Center, New York, NY, USA

## Abstract

At the plenary session of the 2008 annual meeting of the American Society of Clinical Oncology, updated results were presented from a large randomized phase III trial comparing adjuvant radiation therapy (RT) and one cycle of Carboplatin for the adjuvant treatment of Stage I seminoma. Results of this Medical Research Council (MRC) trial led its investigators to conclude that one cycle of carboplatin was equivalent in safety and efficacy and less toxic than RT. In this editorial, the trial's design, statistics, toxicity, and length of follow-up are discussed within the context of historical treatments of this disease. With a 1.3% increase in relapse rate (5.3% with carboplatin vs. 4.0% with radiation), a 3% or greater increase in relapse rate could not be excluded, the primary endpoint of the study. A decrease in second testicular germ cell tumors was observed, but was equivalent to the increase in relapse rate. Acute toxicity was generally less with carboplatin. However, the extent of late toxicity, including late second neoplasms, cannot be evaluated because of the short median follow-up. Carboplatin is not yet a standard of care. Surveillance-based strategies, including risk-adapted policies that limit RT to patients with the greatest likelihood of relapse remain prudent at this time.

At the plenary session of the 2008 annual meeting of the American Society of Clinical Oncology (ASCO), the updated results[1] of a large phase 3 randomized trial conducted by the Medical Research Council (MRC) comparing a single dose of carboplatin (AUC = 7) with radiation therapy (RT) as adjuvant treatment of patients with stage I seminoma were reported. The authors had previously published this trial in 2005, [2] at which time a major criticism was the short median follow-up time of 4 years. Now, with a median follow-up of 6.5 years, 5-year relapse rates of 5.3% for carboplatin and 4% for radiation were reported, with a significantly reduced frequency of contralateral primary testicular tumors (0.3% vs. 1.7%) and dyspepsia, and a greater ability to work 4 weeks after starting treatment in the carboplatin arm.[1] The authors stated that the 1.3% difference in absolute relapse rates between the 2 arms with the sample size of 1477 can reliably exclude ≥ 3.6% higher relapse rate with carboplatin as compared to RT. The authors concluded that "...with mature follow-up, one course of carboplatin is safe, less acutely toxic, and as effective as radiation for stage I seminoma".[1]

Based on these results, should carboplatin now be offered to all patients with stage I seminoma? In order to answer this question, it is necessary to review expected outcomes, the trial's design and statistics, and the toxicity and post-therapy follow-up necessary for the available treatment options.

Stage I seminoma is the most common single stage/histology of testicular cancer, comprising up to 80% of seminomas and 40% of all testicular cancers.[3] Since surveillance (with treatment upon relapse) and adjuvant RT each achieve an overall survival approaching 100%, treatment toxicity, quality of life, and relative intensity of follow-up have dominated discussions of the optimal approach rather than efficacy. Any new treatment option must maintain the negligible disease-specific mortality rate without increasing the frequency of serious adverse events or intensifying follow-up requirements.

The MRC trial was designed to exclude a > 3% increase in relapse rate with carboplatin. The 90% confidence interval for difference in relapse rates was -0.7% to 3.5%, above the predetermined 3% mark. Hence, the study did not meet its primary endpoint. Put differently, with a relapse rate of about 4%, 40/1000 RT patients will relapse and the MRC trial cannot exclude a relapse frequency of > 70/1000 with carboplatin. Moreover, a non-inferiority design is not the same as equivalence, which requires a much larger number of patients. In addition, while the 1.4% decrease in contralateral testicular primaries with carboplatin was statistically significant, it may not be clinically meaningful since it is offset by the 1.3% increase in relapses.

Relapse site is an important consideration. Like surveillance, the majority of relapses after carboplatin occur in the retroperitoneum requiring abdominopelvic CT scans at regular intervals. In contrast, relapses after RT are almost exclusively outside the retroperitoneum (usually thoracic), making such testing unnecessary. The dose of radiation sustained with CT imaging may not be trivial; indeed, a recent publication proposed that such doses could increase the risk of developing a secondary malignancy.[4] The need for post-treatment imaging also requires increased patient compliance compared with RT.

Late toxicity must be also considered. RT, once the standard of care, has recently been shown to significantly increase the risk of secondary malignancies. Two large recent studies show that the risk of secondary cancers is about double that of age-matched controls.[5,6] At particular risk are the 4% of patients who relapse after RT and subsequently require chemotherapy; use of both modalities confers a 3-fold higher risk of developing a secondary malignancy and also significantly increases the rate of coronary disease.[5,6] These risk differentials only begin to emerge after 15 years of follow-up.

Therefore, we feel that 6.5 years median followup (only 25% followed ≥ 8 years) is insufficient to assess the frequency of significant long-term toxicity with carboplatin. This concern is supported by the fact that, to date, a very low frequency of secondary malignancies has been observed in the MRC study with no appreciable difference between the RT and carboplatin arms (1.1% vs. 0.9%). Like RT, chemotherapy (in particular, high-dose carboplatin and standard-dose cisplatin) has been associated with secondary malignancies and cardiovascular disease. [5-7] Whether one dose of carboplatin AUC = 7 might lead to the same or different frequency of late toxicity as historical platinum therapies is unknown, but the lack of long-term data should not be interpreted as proof of safety. While it is encouraging that a recent report[8] noted no increase in deaths, cardiovascular disease, or secondary malignancies after 1 or 2 cycles of carboplatin for Stage I seminoma compared with age-matched controls, the number of patients was small (N = 199) and the median follow-up was still < 10 years.

What about surveillance? Surveillance has become increasingly recommended as the association between RT and second malignancy has emerged. A universal adjuvant approach (either RT or carboplatin) subjects 85%–90% of patients to treatment unnecessarily (80–85% patients would be cured with surgery alone and 4% will relapse despite RT). The main concern with surveillance is patient compliance with follow-up. A recent report noted that 21% of seminoma patients on surveillance were lost to follow-up after a median of 5.5 years.[9] At least 5% of relapses occur after five years, suggesting that non-compliant patients will present with more advanced disease, need more intensive treatment, and experience worse overall outcomes.[10] Finally, the incidence of late relapses after carboplatin remains unknown and might be a concern due to inferior efficacy compared with cisplatin in the metastatic setting.[11,12]

What about a risk-adapted approach? Unfortunately, risk factors for relapse in stage I seminoma have been difficult to identify and validate. Combining 3 surveillance series, Warde and colleagues found tumor size > 4 cm and rete testis involvement to predict relapse (risk 12% for patients with 0, 16% with 1, and 32% with both features)[13] but these factors have not been independently validated. Valdevenito reported that size > 6 cm and rete testis invasion were significant risk factors.[14] The Spanish Germ Cell Cancer Cooperative Group (SGCCCG) was the first to study a risk-adapted strategy, treating pT1 patients with surveillance and pT2 patients with 2 cycles of carboplatin.[15] Since tumor size and rete testis involvement were not criteria for stratification, their correlation with relapse could be assessed in the surveillance arm. However, only rete testis invasion had prognostic value.[15] In a second risk-adapted study, these same authors stratified patients based on rete testis invasion and tumor size > 4 cm; patients with neither factor were observed while the remainder received 2 cycles of adjuvant carboplatin. The relapse rate was 3.3% with chemotherapy and 6% with surveillance, demonstrating the feasibility of a risk-adapted approach.[16] One concern from the latter trial is the lack of data regarding long-term toxicity and relapse with carboplatin.

So, what should you recommend to the next patient you see with Stage I seminoma? The advantages and disadvantages of each approach should always be carefully discussed with patients to enable informed decisions. However, we believe that an active surveillance strategy remains the most appropriate management policy, either observing all patients or limiting RT to those most at risk for relapse. Patients must be able to comply with follow-up and handle the psychological burden of not receiving active treatment.

We agree with others[16] that ideal practice would use a validated risk-adapted approach similar to that employed for stage IB nonseminomatous germ cell tumors, thus limiting intervention to the patients with the highest risk of relapse. Employing the international meta-analysis[13] and the Spanish prospective study[16] as a treatment guide, the use of size > 4 cm and rete testis involvement as clinical features identified a high-risk population for whom clinical intervention over surveillance was favored. Approximately 16% of Stage I seminoma patients[16] will fall into this group with an expected relapse rate of 32% [13] if untreated. In contrast, patients with neither of these factors had a relapse rate of only 6% in the SGCCCG trial.[16] Using RT only for the highest risk patients can avoid immediate treatment (and potentially unnecessary toxicity) in 84% of men exclusive of non-compliant patients or those who can not psychologically tolerate surveillance. We view carboplatin as a non-standard alternative when radiation and surveillance are contraindicated.

To eliminate the controversy over treatment for stage I seminoma, risk factors for relapse should be further refined; clinical factors such as age[13,16], pre-orchiectomy HCG[16] and microarray signatures may be of benefit in identifying high risk patients. Our approach at Memorial Sloan-Kettering Cancer Center utilizes a risk-adapted approach, recommending RT if the primary tumor is > 4 cm AND rete testis invasion is present, with a research emphasis on identifying clinical and biological factors that can refine high risk criteria. Collaborative efforts to validate high risk features prospectively will be essential to optimizing our ability to identify seminoma patients warranting immediate intervention.
